# The Value of Interdisciplinary Collaboration in Human Geography and Bioethics

**DOI:** 10.12688/openreseurope.17045.1

**Published:** 2024-03-04

**Authors:** Daniel P. Jones, Kristien Hens

**Affiliations:** 1 Newcastle University, Newcastle upon Tyne, England, NE1 7RU, UK; 2 Philosophy, Universiteit Antwerpen, Antwerp, Flanders, 2000, Belgium

**Keywords:** human geography, interdisciplinarity, transdisciplinarity, bioethics

## Abstract

Human geography and bioethics both take pride in their interdisciplinary approaches. Relatively little cross-pollination has occurred between human geography and bioethics. This paper takes three cases to highlight the generative potentials of both disciplines, dedicating time and space to learning from each other. Through doing so, we highlight these potentials by focusing on how navigating public spaces subverts the expected uses of particular spaces. We demonstrate that these are entangled with questions of responsibility that both geographers and bioethicists might find helpful. Human geographers and bioethicists can, and should, look for non-naïve ways to care for *space,* and we hope for this paper to be an example of where to start in the collaborative future of our disciplines.

## Introduction: bioethics and space

Bioethics is a field that reflects on the duties of medical professionals and researchers vis-à-vis their patients and research participants, and also often on the development of new technologies, such as cloning or CRISPR/Cas9. Apart from these ‘mainstream’, usually ‘principlist’ approaches, in the eighties and nineties, scholars advocated for bioethics that was grounded more in feminist care ethics and narrative ethics. In the last decades, and with the acknowledgement that health and environmental ethics are intrinsically linked, bioethicists have sought to incorporate ideas from disability studies, postcolonial ethics and environmental ethics.

Bioethics has always claimed to be interdisciplinary ^1^. Bioethicists have different backgrounds: some are philosophers, some are lawyers, and some are medical doctors. Moreover, bioethics scholars often work closely with biomedical researchers and clinicians due to the subject they study. At the same time, this cooperation is not always felt to be two-sided: bioethicists are often invited on big research projects at the last minute to fill in the obligatory ‘ethics’ part that many funding agencies require. As such, there is little to contribute to the design of the project or the concepts that the project uses. Ethics is an afterthought. Bioethicists also often borrow methodologies from other social sciences and humanities fields or acknowledge the potential for using arts to illustrate bioethical findings and issues without considering what the arts, humanities, and social sciences can contribute to producing those very findings ^2^. Indeed, the ’empirical turn’ in bioethics has made many bioethicists look into sociological and anthropological methods such as interviews, surveys and ethnographical work to explore ethical dilemmas in practice ^3^. Given the increased acknowledgement of the entanglement of human beings and their milieu, bioethicists have come to acknowledge that ‘space matters’ for health. The recent interest in epigenetics is a case in point. Whereas many bioethicists have long studied the ethical implications of genetic knowledge and genetic technologies, the advent of postgenomic science has challenged ethicists to let go of atomistic views on human beings. Social, cultural and physical context matters for health and, hence, for ethics. In 2003, Susan Kelly argued that “Being able to think about place and practice in clear yet theoretically nuanced ways may provide an important antidote to a well-recognised shortcoming of modern bioethics” ^4^. From that viewpoint, bioethical reflection can significantly benefit from insights from human geography.

At the same time, borrowing some other discipline’s methods is not the same as interdisciplinarity. We believe that an engagement in the methods of a particular field also implies engaging with the theoretical underpinnings of that field. In what follows, we explore how such ‘true’ interdisciplinarity between bioethics and human geography can take shape. We elaborate on ideas we have first put forward in an editorial of the journal *Bioethics ^5^*.

## Introducing Human Geography

Human Geography, as a subdiscipline within the field of geography, is often misunderstood in its focus. As per the words of Gibson, “[a]n outsider could be forgiven for thinking that human geography was the study of the existence and distribution of humans on Earth” ^6^. Perhaps it is difficult to define due to the diverse nature of its contents, from economics to disability to social policy. It was first used as a term in the 1920s within academic literature to talk about nature and human debates, and it evolved into considering human affairs in the 1950s ^7^. With that in mind, generally speaking, the goals or aims of Human Geography as a discipline are to examine the space, place and scale, or the processes that happen within spaces and places that affect or involve humans; space, place and scale are concepts that are significant to consider within the discipline of human geography, as it allows explicitly scholars to think *spatially*. Practically, this looks like a deep and critical consideration of location, condition, connections and scale. Geographers consider location *a position in space* because seeing something’s position in space allows you to make comparisons, considering different conditions and contexts. Moreover, human geography takes a significant interest in *place*, distinct from simply *location*. Place comprises various conditions: environment, history, politics, economy, and culture. By taking these conditions of a location into account, we start to build an understanding of a *place* with meaning; the *place* is made up of *stuff*, inclusive of processes and relations: it is not static, but rather “a moment’s constellation of social relations” ^8^. Take Newcastle-upon-Tyne as one example – a northeastern UK coastal city (location), which influences its economic history of industry and shipping ports. The conditions shift through processes such as deindustrialisation that have led to the changing of primary working sectors and cultural identities of the city; once known for industry, it now has a reputation as a thriving nighttime economy for students and young adults ^9^. Once conditions of a place are outlined, geographers consider the connections, be they natural or artificial. When we look at the links between spaces and consider just how intertwined they are with each other, we can make comparisons that consider region and scale.

Whilst the aforementioned links could be physical such as a river or a road that links two locations together, they could also be immaterial. These could be places not necessarily linked by river or land, as two examples, but rather by other means. For example, an organisation may engage in the toilet-twinning scheme ^10^, whereby one organisation pays for the construction of a toilet in another community. They may be thousands of miles apart, but the connection remains through this imagined connection. The same might be said for places where a corporation has designated international offices, places that are connected by a musician who tours across a country in different cities, or even on a personal scale of having family members dotted across the globe. This imagined connection consists of some kind of meaning or emotional attachment to a place, be it in familial ties or of a diasporic nature.

Ultimately, human geography is about people and their relationships to their surroundings, inclusive of the human and other-than-human. It involves human interaction with environments, meaning that it is inclusive of political, cultural, and organisational aspects of being. Within this falls also an interest in health, and this application of the discipline can be traced back to the cholera outbreak of 1854 in London, UK. Dr John Snow began plotting infection sites on a map, enabling him to notice a cluster of cholera cases around one specific water pump, which was later found to have been contaminated. The pump was disabled, and cholera cases immediately began to decline ^11^. In some ways, this was the start of a deeper consideration of human geography; today, health geography is a much more significantly developed field. In the words of Curtis & Rivera, “Geographical research on human health and diseases is concerned with the processes and relationships in space and time that govern human interactions with their environment and with each other, in complex and ‘non-linear’ ways” ^12^. This could be in a variety of ways, including but not limited to considerations of climate change and globally contrasting geographies of health and wellness, the relational geographies of disability, hate crime and belonging in urban city spaces ^13^, or intersectional approaches within COVID-19 discourse regarding the production of anti-oppressive research outcomes, more generally speaking ^14^.

The relevance and importance of lived experience accounts are acknowledged with respect to health geographies ^15^. It has been written that following relational geographies, considering place not as containers of process and social relations but rather as made up of them, the human geographies of health begin to consider place not as bounded or static but as “a moment’s constellation of social relations” ^16^. These moments’ constellations arguably share thematic interests with the discipline of bioethics.

## On interdisciplinarity

Interdisciplinarity has become somewhat of a buzzword within academic spaces in recent years. Ironically it seems that as we silo ourselves into singular disciplines as academics, we develop differing understandings of what it means to be interdisciplinary in our work. Is it collaborating and co-authoring across disciplines, perhaps like we are doing with this paper, or is it learning and applying concepts and ideas that we think might work for our research from areas outside of our discipline whilst not engaging with others? Whilst we, as bioethicists and geographers here, might claim and aim to be interdisciplinary in our approach to our research interests, do bioethicists know what human geography is? Do human geographers know what bioethics is? This collaboration resulted from a research visit whereby Daniel visited the *NeuroEpigenEthics* project at Antwerp University. Initially, from Daniel’s geographer’s perspective, bioethics predominantly biomedical ethics, just like him; before the visit, Kristien’s understanding of human geography was that it dealt with issues such as population density, transport and urbanisation quantitatively. However, through spending time dedicated to learning from each other and our respective disciplines, this process of *interdisciplining* of sorts has offered wonderful insights into just how much we don’t know about other disciplines and the sheer potential for nuanced and groundbreaking insights that true interdisciplinarity between our disciplines can offer. Ultimately, we want to echo Olson’s paper that reads, “It shouldn’t take us 20 more years to convince moral philosophers and ethicists that space is not inert, and geography is more than a metaphor” ^17^. Olson is correct. The time is now for meaningful and purposeful interdisciplinarity; it is time to move beyond our disciplinary pride as academics.

A search for peer-reviewed publications on interdisciplinarity, bioethics and human geography might raise questions of whether or not this has been done before and what the point of this interdisciplinarity might be. We aim here to highlight that interdisciplinary collaboration within research is not limited to research outputs, nor should it exclusively start with bioethics as per Couture *et al.*’s call ^18^ (nor vice versa). Indeed, human geographers, just as all researchers who study humans, need to conduct their research ethically and can engage bioethicists to help with issues related to research participation, privacy and consent. However, we argue that true interdisciplinarity can occur within various research projects' knowledge-production stage(s). However, we seek to move beyond simply bioethics helping with the ethics of doing research and wish to consider the deeper generative ways collaboration will inform each discipline.

Smith ^19^ writes about the meeting point between radical geography and normative theory, and we believe that this is evidence that there is a need and value to foster intentional interdisciplinarity between bioethics and human geography, as disciplines that have previously been wary of each other. There have previously been attempts to *bridge the gap* between critical bioethics and the social sciences, particularly highlighting the gap that can often exist between “bioethics and what goes on in the clinic” regarding the difference in bioethicist accounts and those who have lived experience or who have spent time in those clinics, so to speak. However, papers like this often refer to incorporating one discipline into the other rather than actively being interdisciplinary in approach (and knowledge exchange). So, what does it mean to be interdisciplinary? We cannot claim to be interdisciplinary if we pick and choose elements we like the sound or feel of; interdisciplinarity involves sitting with the tensions as well and working with and through them.

Whilst there has been progress in applying ways in which bioethics and human geography can learn from each other, we believe something is missing. Carvalho, Shimizu & Garrafa ^20^ approached the work of Josué de Castro, specifically the works titled *Geographies of Hunger* ^21^ and *Geopolitics of Hunger*, ^22^ from a bioethical perspective, acknowledging themselves that whilst Castro never specifically worked within the bioethical sphere, bioethics could learn from it – through doing this, the authors noticed some similarities between the geographical work of Castro and their own work as bioethicists in thinking through hunger and social justice. Meanwhile, McCurdy ^23^ approaches the work of geographer Katherine McKittrick’s “Demonic Grounds: Black Women and the Cartographies of Struggle” ^24^ in an attempt to illustrate bioethical eurochristian biases, assumptions and general thinking that are present within the field of bioethics due to bioethics being a “product of dominant ‘eurochristian’ worldview” ^25^. Whilst these are both papers that share essential insights and criticisms, what appears to be missing here is having bioethicist-geographer collaboration from the outset being intended, beginning with the research design. There seems to be work of bioethicists using geographers’ work, and vice versa. Still, it is to our knowledge that bioethicists and geographers have not explored the true collaborative potential of human geography and bioethics as a team.

With this in mind, this paper defines interdisciplinarity as a genuinely collaborative and iterative process through which the starting point is not one discipline or another but rather cases or instances or occurrences. We use the following cases to suggest ways in which our disciplines can learn from and teach each other in a truly interdisciplinary and collaborative process of knowledge generation. In doing so, we hope to demonstrate that ethics is truly everywhere, embedded deep within disciplines, and perhaps that we just haven’t noticed it in the first instance…

## Case 1: Parking Spaces – Navigating rights and duties in public spaces

For this first case, we wish to discuss work surrounding parking spaces. The parking space, found on the side of a road or perhaps in a designated parking lot, is something that is frequently a paid-for-space, oftentimes charged at an hourly rate, and that is usually reserved for cars or other motorised vehicles such as motorbikes, mopeds, vans, etc. For bioethicists, questions might arise surrounding disability policy, questions over what an inclusive society might imply and how to distribute goods such as parking spaces in a way that is *just ^26^*. For human geographers, questions arising might include but not be limited to questions over the critiquing of on-street policies for street and cycling safety ^,^ experiences had by those who use disabled parking bays, or perhaps even creative ways of subverting the expected use of a paid-for parking space through the hosting of parking space picnics and protests ^27^. There are evidently some overlaps that make immediate sense here, such as interests in policy and distribution in the case of parking spaces. However, the latter example of creative and subversive uses of public spaces (in which parking spaces are often found) are things we argue that human geographers and bioethicists alike can learn from each other within inquiry.

**Figure 1.**
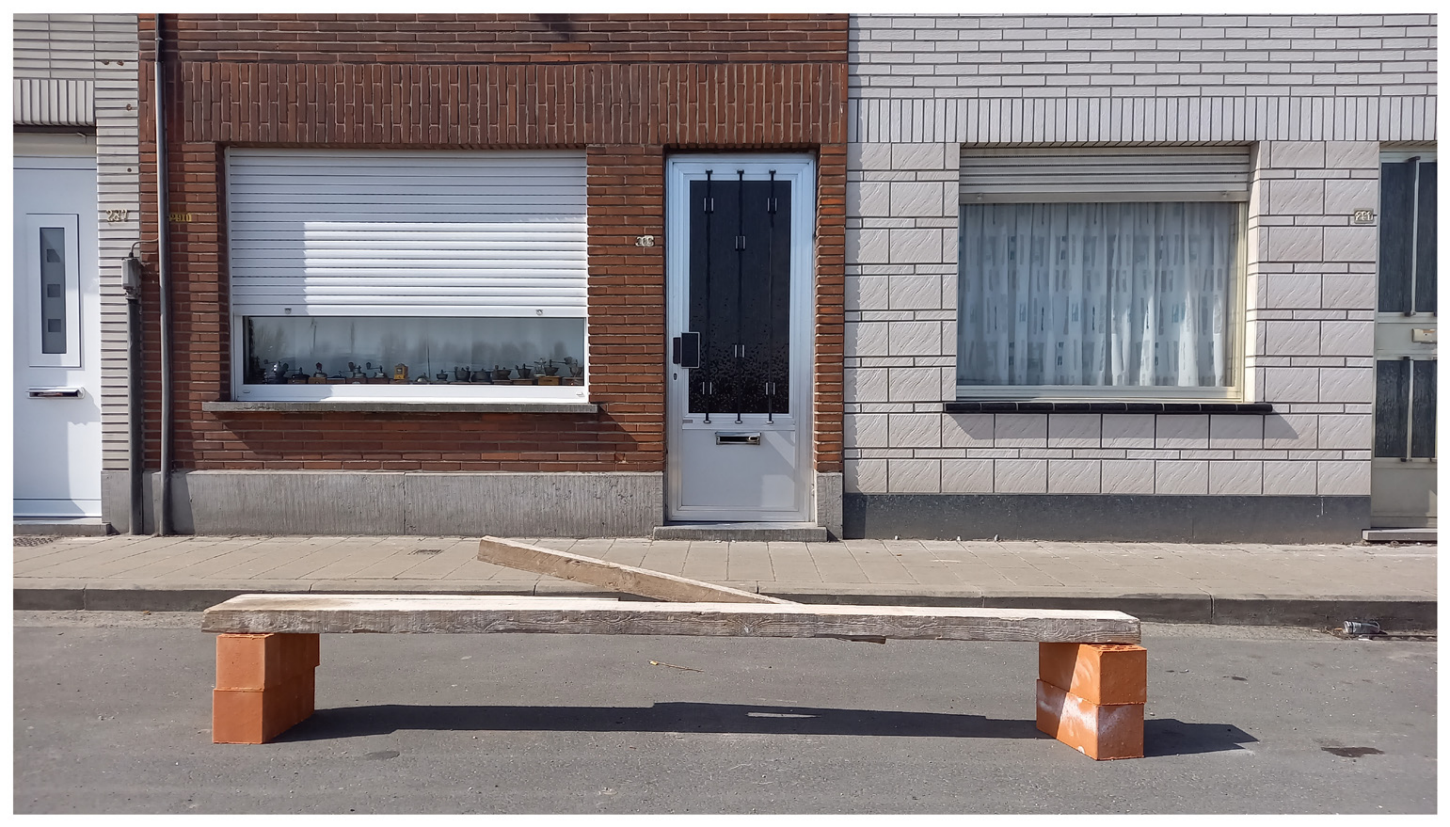
An image from Anything Can B_ A Car, showing an improvised bench made of bricks and a wooden plank in a parking space outside of a house (Image © Karel Verhoeven, reproduced with permission from Nele Buyst. Further reproduction is not permitted without prior agreement with the copyright holder).

The latter, in particular, is explored by artists and performers alike, such as in the photographic series by Karel Verhoeven, *Anything Can B_ A Car ^28^.* This project shows the alternative uses of parking spaces for placing sculptural objects such as chairs and artwork in the streets for various reasons. As the project develops, the collection of images is developing into an archive that looks to “map human behaviour in relation to public space and indicates a certain level of intuitive artistic practice”, with Verhoeven being particularly interested in the creative human uses of public spaces and the placement of temporary public artefacts that may be personal and intimate to those who place the bench, for example, on the side of the road ( Figure 1).

Policies regarding the accessibility of parking spaces are part and parcel of the ethics of disability. What can be learnt from human geography is the impact and experiences of and in spaces and why that matters. The consideration of everyday subversion of public spaces and mundane creativities is something that has been explored in depth within human geography, whether in the aforementioned examples of Sachs Olsen or in thinking about the creative responses to everyday affective events that may be passed off as otherwise *mundane ^29^.* Perhaps then, what the bioethicist might be able to learn here is precisely the importance of moving beyond the unintentional taking for granted of the ‘givenness’ of spaces, be it in what or who is occupying or owning them or the ways in which they are used. Perhaps, sometimes, bioethics takes these things too much for granted.

On the other hand, the human geographer could benefit from the bioethical approach of returning to the normative aspects of inquiry within the consideration of everyday, mundane and vernacularly creative uses of the parking space. What are the practical implications of alternative use of a parking space other than its intended service provision of *a place to store the car*? What are the questions of morality and responsibility at play? What are the assumed terms of service that influence the right to use the parking space(s) in question, and how do these terms of service influence assumptions regarding ‘normal’ or ‘appropriate’ behaviour in and around them? Further to this, a collaborative bioethical and geographical approach may also raise questions beyond these, such as that of environmental morality in thinking about how the use of parking spots for arts and protest shows a moral decision in the limitations of vehicular traffic in a given area, and therefore a limitation of CO2 emissions in the area, in turn playing a role in pushing back against climate change and its associated consequences for humanity.

## Case 2: Parkour – learning from playful experiences

With the aforementioned in mind, we here seek to expand our consideration into the case of parkour. With its origins in suburban Paris, David Belle began to use skills learnt in martial arts and gymnastics to explore and' trace’ the suburbs, “overcoming any physical objects via athleticism and flexibility, usually jumping, climbing, and running” ^30^. Parkour is particularly interesting from a human geographer’s perspective in its creative reimagining of the ways in which the built environment can be used; for example, how might a series of bollards enable above-ground movement between sides of a street in the practice of parkour, rather than its desired effect of restricting the mobility in the area via vehicular means such as with cars and vans that cannot fit through the gaps between each bollard? Perhaps those geographers interested in commodities may also ask about the commercial adoption of parkour and free-running in advertising vehicles, sportswear, etc.

Bioethical interests are also peaked here in thinking about questions of specific sporting practice, insurance, and asking the question of who exactly is responsible for the injury that may occur in the sporting practice of parkour, given that it has been officially recognised as a sport in countries such as the UK (as of 2017). Indeed, bioethicists have engaged in reflection about the ethical aspects of sports in general, for example, on the topics of the use of doping. As parkour becomes increasingly mainstream, including tournaments sponsored by specific brands or advertisements of sportswear and vehicles using parkour, existing ethical discussions about extreme sports and their use in advertising may offer insight into the class geographies of parkour, considering who has access to insurance for extreme sports; gendered representations of parkour; aspects of stigma within extreme sports, and so on.

Parkour is a way of appreciating buildings and structures that are often taken for granted. Many able-bodied people do not pay much attention to the steps in a railway station, rails or fences. Viewed through the lens of parkour, they become obstacles or aids, much in the same way as the forced creativity required for disabled people to get from A to B ^31^. Bioethics has often been accused of being ableist and taking for granted that what we should strive for is the eradication of disability, through genetic and prenatal testing. However, in the last decades, disability bioethics has taken flight ^32^. These approaches, often in tandem with feminist bioethics, have argued for the value of different types of minds and bodies and have advocated for the inclusion of experiences of disabled people in bioethical reflection. Such an engagement can be vastly enriched if experiences of the use of space are taken into account. What does public space convey about who is welcomed in a specific society? What norms are engrained in public space, and are these norms acceptable if we take a care-full disability ethics stance? What kind of creativity can we expect from people for whom the standard organisation of public space poses obstacles? Maybe the example of parkour and geographer’s reflections on what this conveys about space can offer valuable insights about how to think about how much creativity we may expect from disabled people, but also how much creativity is owned to them in the design of public spaces.

The creative engagement of parkour with public spaces may also inspire a broader conception of care. Bioethicists have embraced care ethics as an alternative or addition to principlist approaches to bioethics. However, they have primarily used it in the context of human relationships, more specifically the relationship between caregiver and care-receiver. At the same time, authors such as Maria Puig de la Bellacasa have described a posthuman ethics of care that defines care as a concrete work of maintenance of our world ^33^. Indeed, if we conceptualise human beings as interdependent and entangled with the more-than-human world, care equally applies to the public space around us. Human geographers and bioethicists could think together about what such caring for space may entail. Thinking *with* parkours as a reclaiming of neglected spaces can make the need for caring for such spaces more salient. We believe a posthuman concept of care, one that includes not only caring for animals or even plants but everything that is part of the built and non-built world, is a promising one to rethink relations with space and technology. At the same time, this is far removed from everyday bioethical practice. We believe having bioethicists and human geographers engage in meaningful conversation can pave the way for an inclusive concept of care.

## Case 3: Health and Responsibility – entanglements of the biological and the social

A salient example of how bioethics and human geography can work together is the question of air quality in buildings. Geographers have reflected on pollution, sustainability, and health, as they are significant aspects across all sub-disciplines of geography, whether environmental or human in nature. Also, geographers have questioned the impact of quality of life and pollution on individuals’ mobilities and living practices within a city or urban environment. Here, questioning presumed intended use, ownership and the entanglement with the social sphere can help preexisting questions about who is responsible for air quality. Bioethicists have asked questions about the attributions of responsibility in the context of pollution, epigenetics and air quality…

Biomedical ethics and environmental ethics have traditionally evolved as separate disciplines, although Van Rensselaer Potter, who was one of the first scholars to use the term ‘bioethics’, advocated for a bioethics that would encompass both the medical and the environmental sphere ^34^. One of the key areas that both human geographers and bioethicists can tackle together is the recent problematisation of the concept of environment. In the last decades, findings in epigenetics and other postgenomic sciences have corroborated the idea that health and the environment are intertwined. It is well known that epigenetics demonstrates the molecular link between individual biology and environment in the broadest sense: particulate matter, psychological experiences, and social habits resonate molecularly. Thus, the naïve idea of place as something that surrounds us is challenged, a thought that is already well acknowledged by human geography as a field. Perhaps even more challenging for both human geography as well as bioethics is the concept of the holobiont, and the idea that we are the environment for microscopic creatures such as bacteria that may define us from the inside out.

Also, pandemics such as the COVID-19 pandemic have been linked with environmental issues such as biodiversity loss ^35^. Moreover, living in buildings with bad air quality can exacerbate the effects of viral infections. For example, Laura Menatti and colleagues discuss the situatedness of health in the case of the pandemic ^36^. They quote Morawska *et al.*: “For decades, the focus of architects and building engineers was on thermal comfort, odour control, perceived air quality, initial investment cost, energy use, and other performance issues, whereas infection control was neglected” ^37^ and explain how the pandemic has raised awareness of the impact of the design and management of built environments. They state: “The case of microbiologically healthier buildings is another example of an adaptive mechanism involving the environment, which could constitute a step forward in coping with COVID-19 and future epidemics and pandemic events”. At the same time, this insight does not give a straightforward answer to the questions of who is responsible for the air quality and what can be done about it. Is the owner of the house or its tenants responsible? The architect weighs several factors, including isolation and the high cost of heating when access to specific fuel sources is limited due to geopolitical events. Indeed, it is all well to say that ‘we’ have a responsibility to provide good air quality, but an essential aspect of this question is related to the space itself and how we conceive it. This is even more so amidst changing landscapes of working and studying from home following the COVID-19 pandemic. In prepandemic times, a classroom was for many different from a living room, which is yet different from an office space or a factory in terms of who is responsible for providing good air quality. It may be helpful to get inspiration from those practices and cultures in which the specific roles and uses of certain spaces have always been mixed. 

At the same time, understanding the dynamics of power and responsibilities to the extent that it is possible to suggest fair policies requires understanding them in the context of the meaning of actual spaces and the relationships with the people in them. For example, how does a schoolteacher navigate the dynamics in their classroom when there is a general recommendation to keep windows open as much as possible while respecting the need for warmth of some of the students? How do abstract guidelines translate into the concrete circumstances of tenants who are dependent on house owners for improvements? Working together with human geographers can help bioethicists take space seriously and get an insight into the social aspects of who gets to live where. Human geography has studied the geographies of class extensively. For example, social housing is a key theme within the discipline. Together, human geographers and bioethicists can make apparent the links between actual spaces and specific responsibilities linked to humans’ situatedness in space.

## Conclusion

In our editorial ‘Ethics is everywhere: Human Geography, Bioethics and the value of interdisciplinary collaboration’ in the journal Bioethics, we argued that *Human geographers can learn from bioethicists about the normative impact of discussions on biology and the nature/culture divide. Bioethicists can learn from human geographers about the normativity of entanglement with space itself. Collaborating from the outset, then, can provide new and nuanced insights for the sector as a whole.* and. Who gets to live under which circumstances” is an ethical question of increasing importance. *disciplines as seemingly far apart as human geography and bioethics can, and should, look for non-naïve ways to care for space that take into account the specificity of contexts and circumstances and help to acknowledge these cases and questions in the view that ethics is truly everywhere.* In this article, we provided some substance to that claim by elaborating on three cases. First, thinking with parking spaces enabled us to on the one hand engage with rights and duties, which is the realm of the bioethicists, and with power implications of the decisions in the public space, which is the realm of the human geographer. Second, thinking with parkours, we described the importance of engaging playfully with experiences of place, the normative implications of such playful engagement and of gaining insight into how the way we value certain bodies reflects space. The third case, epigenetics, is a demonstration of how ‘place’ can become ‘biology’. Human geographers can learn from bioethicists about the normative impact of discussions on biology and the nature/culture divide. Bioethicists can learn about the normativity of entanglement with space itself from human geographers.

With these cases and this paper, we hope to have sketched a joint future of human geography, bioethics, and interdisciplinary work in general. 

## Data Availability

No data are associated with this article.

## Footnotes

^1^ Ives, J. (2014). A method of Reflexive Balancing in a Pragmatic, Interdisciplinary and Reflexive Bioethics. *Bioethics, 28*(6), 302–312.

^2^ Couture, V., Beisle-Pipon, J., Cloutier, M & Barnab, C. (2017) Merging arts and bioethics: An interdisciplinary eaxperiment in cultural and scientific mediation. *Bioethics, 31*(8), 616–630.

^3^ Hoffmaster, B. (1992). Can ethnography save the life of medical ethics? *Social Science & Medicine (1982)*. *35*(12), 1421–1431. https://doi.org/10.1016/0277-9536(92)90045-r THE BIRTH OF THE EMPIRICAL TURN IN BIOETHICS - BORRY - 2005 - Bioethics - Wiley Online Library. (n.d.). Retrieved 31 May 2023, from https://onlinelibrary.wiley.com/doi/abs/10.1111/j.1467-8519.2005.00424.x

^4^ Kelly, S.E. (2003). Bioethics and rural health: theorizing place, space, and subjects. *Social Science & Medicine*. *56*(11), 2277–2288. https://doi.org/10.1016/S0277-9536(02)00227-7

^5^ Jones, D. P., & Hens, K. (2023). Ethics is everywhere: Human Geography, Bioethics and the value of interdisciplinary collaboration. Bioethics. https://doi.org/10.1111/bioe.13209

^6^ Gibson, C. (2010). Guest editorial-creative geographies: Tales from the ‘margins’. *Australian Geographer, 41*(1), 1–10.

^7^ Gibson, C. (2010). Guest editorial-creative geographies: Tales from the ‘margins’. *Australian Geographer, 41*(1), 1–10.

^8^ Prior, L., Manley, D. & Sabel, C. (2019). Biosocial health geography: New ‘exposomic’ geographies of health and place. *Progress in Human Geography, 43*(3), 531–552. p535.

^9^ Chatterton, P., & Hollands, R. (2002). Theorising Urban Playscapes: Producing, Regulating and Consuming Youthful Nightlife City Spaces. *Urban Studies*. *39*(1), 95–116.

^10^ Cadée, F., Nieuwenhuijze, M.J., Lagro-Janssen, A.L.M., & De Vries, R. (2016). The state of the art of twinning, a concept analysis of twinning in healthcare. *Globalization and Health*. *12*(1), 66. https://doi.org/10.1186/s12992-016-0205-5 Zelinsky, W. (1991). The Twinning of the World: Sister Cities in Geographic and Historical Perspective. *Annals of the Association of American Geographers*. *81*(1), 1–31.

^11^ Snow, J. (1856). On the mode of communication of cholera. *Edinburgh medical journal, 1*(7), 668.

^12^ Curtis, S. & Riva, M. (2010). Health geographies I : Complexity theory and human health. *Progress in Human Geography, 34*(2), 215–223. p217

^13^ Hall, E. & Bates, E. (2019). Hatescape? A relational geography of disability hate crime, exclusion and belonging in the city. *Geoforum, 101*(March), 100–110.

^14^ Eaves, L. & Al-Hindi, K. F. (2020). Intersectional geographies and COVID-19. *Dialogues in human geography, 10*(2), 132–136

^15^ Kears, R. A. & Moon, G. (2002). From medical to health geography: novelty, place and theory after decades of change. *Progress in Human Geography, 26*(5), 605–625

^16^ Prior, L., Manley, D. & Sabel, C. (2019). Biosocial health geography: New ‘exposomic’ geographies of health and place. *Progress in Human Geography, 43*(3), 531–552. p535.

^17^ Olson, E. (2018). Geography and ethics III: Whither the next moral turn? *Progress in Human Geography. 42*(6), 937–948. P937.

^18^ Couture, V., Bélisle-Pipon, J.-C., Cloutier, M., & Barnabé, C. (2017). Merging arts and bioethics: An interdisciplinary experiment in cultural and scientific mediation. *Bioethics*. *31*(8), 616–630. https://doi.org/10.1111/bioe.12391

^19^ Smith, S. (2009) Everyday morality: Where radical geography meets normative theory. *Antipode*, 206–209.

^20^ Carvalho, L., Shimizu, H. & Gaffara, V. (2019) Geography and geopolitics of hunger: bioethics in the workd of Josue de Castro. *Revista Bioetica 27*(1), 143–152.

^21^ Castro, J. de. (1952). The Geogra De Castro, J. (1952). *The geography of hunger* (Vol. 74, No. 5, p. 404). LWW.phy of Hunger. Little, Brown.

^22^ Castro, J. de. (1977). The Geopolitics of Hunger. Monthly Review Press.

^23^ McCurdy, J. (2022) Colonial Geographies, Black Geographies, and Bioethics. *Hastings Center Report 52*(S1), S66–S68.

^24^ McKittrick, K. (2006) *Demonic grounds: Black women and the cartographies of struggle.* University of Minnesota Press.

^25^ McCurdy, J. (2022) Colonial Geographies, Black Geographies, and Bioethics. *Hastings Center Report 52*(S1), S66–S68.

^26^ Litman, T. (2002) Evaluating transportation equity. *World Transport Policy & Practice, 8*(2), 50–65.

^27^ Sachs Olsen, C. (2018). Collaborative challenges: Negotiating the complicities of socially engaged art within an era of neoliberal urbanism. *Environment and Planning D: Society and Space, 36*(2), 273–293.

^28^ Verhoeven, K. (2014). *Anything Can B_ A Car*. Retrieved from https://www.karelverhoeven.be/projects/anything.html

^29^ Edensor, T., & Millington, S. (2018). Spaces of vernacular creativity reconsidered. In *Creative Placemaking*, 28–40. Routledge.

^30^ Mould, O. (2009) Parkour, the city, the event. *Environment and Planning D: Society and Space. 27*(4), 738–750. p 738

^31^ Butler, R., & Bowlby, S. (1997). Bodies and Spaces: An Exploration of Disabled People’s Experiences of Public Space. *Environment and Planning D: Society and Space*. *15*(4), 411–433. https://doi.org/10.1068/d150411

^32^ Vanaken, G.-J. (2022, April 26). Cripping vulnerability a disability bioethics approach to the case of early autism interventions. PsyArXiv. https://doi.org/10.31234/osf.io/jerhk Reynolds, J.M., & Wieseler, C. (2022). The Disability Bioethics Reader. Taylor & Francis.Scully, J.L. (2008). Disability Bioethics: Moral Bodies, Moral Difference. Rowman & Littlefield.

^33^ Bellacasa, M.P. de la. (2017). Matters of Care: Speculative Ethics in More Than Human Worlds. University of Minnesota Press.

^34^ Potter, V. R. & Whitehouse, P. J. (1998) Deep and global bioethics for a livable third millennium. *The Scientist, 12*(1), 9.

^35^ Lawler, O.K., Allan, H.L., Baxter, P.W.J., Castagnino, R., Tor, M.C., Dann, L.E., … Kark, S. (2021). The COVID-19 pandemic is intricately linked to biodiversity loss and ecosystem health. *The Lancet Planetary Health*. *5*(11), e840–e850. https://doi.org/10.1016/S2542-5196(21)00258-8

^36^ Menatti, L., Bich, L. & Saborido, B. (2022). Health and environment from adaptation to adaptivity : a situated relational account. *History and Philosophy of the4 Life Sciences, 44*(3), 1–28.

^37^ Morawska, L., Allen, J., Bahnfleth, W., Bluyssen, P. M., Boerstra, A., Buonanno, G., Cao, J., Dancer, S. J., Floto, A., Franchimon, F. & Greenhalgh, T. (2021). A paradigm shift to combat indoor respiratory infection. *Science, 372*(6543), 689–681.
