# Supplementary material for: The Value of Interdisciplinary Collaboration in Human Geography and Bioethics
Source: Open Res Eur. 2024 Mar 4;4:49. [Version 1] doi: 10.12688/openreseurope.17045.1 (PMC11128043; doi:10.12688/openreseurope.17045.1)
Supplement: Peer review history

# Reviewer report: Reviewer response for version 1

**DOI:** 10.21956/openreseurope.18419.r39690

**Published:** 2024-05-25

**Authors:** Michiel De Proost

**Affiliations:** 1 Bioethics Institute Ghent, Department of Philosophy and Moral Sciences, Ghent University, Ghent, Belgium

This is a well-written and interesting essay that sketches out how interdisciplinarity work between bioethics and human geography can take shape. An idea that the authors have launched in an editorial of the journal *Bioethics* but further substantiated in this essay by elaborating on three cases: 'parking spaces', 'Parkour', and 'the question of air quality in buildings'.

 I have two suggestions that the authors may wish to consider regarding their paper:

- Care ethics is described several times as an alternative or addition to principlism. However, it is not clear how feminist care ethics, and the values associated with it, are a more appropriate normative framework to address patterns of responsibility. I think the work of Margaret Urban Walker, especially her notion of "geographies of responsibility" could be relevant here.
- It is also not clear how the interdisciplinary collaboration in human geography and bioethics relates to the "empirical turn" in bioethics. In the latter field, the requirement to integrate empirical research and normative argument is still controversial. Referring to the debate on standards of empirical bioethics (cf. Ives et al., 2018) among other work, could maybe be of added value.

Minor comments:

- The format of the references should be checked. See for instance: Bellacasa, M.P. de la. (2017). Matters of Care: Speculative Ethics in More Than Human Worlds. University of Minnesota Press.
- Further proofreading needs to be done to catch minor grammar/syntax issues.

Is the case presented with sufficient detail to be useful for teaching or other practitioners?

Yes

Is the work clearly and accurately presented and does it cite the current literature?

Yes

If applicable, is the statistical analysis and its interpretation appropriate?

Not applicable

Are all the source data underlying the results available to ensure full access and reuse by other researchers?

No source data required

Are the conclusions drawn adequately supported by the data and analysis?

Yes

Is the background of the case’s history and progression described in sufficient detail?

Yes

Reviewer Expertise:

Feminist empirical bioethics

I confirm that I have read this submission and believe that I have an appropriate level of expertise to confirm that it is of an acceptable scientific standard.

# Reviewer report: Reviewer response for version 1

**DOI:** 10.21956/openreseurope.18419.r38877

**Published:** 2024-05-08

**Authors:** Soren Holm

**Affiliations:** 1 University of Manchester, Manchester, UK; 2 University of Oslo, Oslo, Norway

This is only a 'case report' in the extended sense of that term, so not all the set questions apply.

 The analysis of what human geography and bioethics can mutually offer each other is illuminating and the conclusion that true interdisciplinary collaboration is important is well substantiated.

 I have two queries about the paper, which are both queries for some further explication.

 1. The central point of contact identified and discussed in the paper is around the concept of 'space'. Should that have been more prominent in the title of the paper?

 2. The link between epigenetics, bioethics, and a deeper understanding of space is mentioned several times in the paper, but I don't think it is fully and convincingly explicated anywhere. Could a critic of this part of the argument not say something like 'Bioethics has often worked with a bio-psycho-social model of health and disease where each of the three domains influence the other two. It has never been denied that the environment influences the body and the development of the body. Epigenetics does not add anything qualitatively new, just another mechanism for that interaction.'?

 I also have some minor comments

 a. On page 3 it is implied that ethicists held 'atomistic views on human beings' before the advent of postgenomic science. Is there any evidence that all or even most ethicists held such views? It is probably true that some did, but is there evidence for the stronger claim?

 b. On page 4 something is missing in the sentence beginning 'Initially, from Daniel's geographer's perspective....

Is the case presented with sufficient detail to be useful for teaching or other practitioners?

Yes

Is the work clearly and accurately presented and does it cite the current literature?

Yes

If applicable, is the statistical analysis and its interpretation appropriate?

Not applicable

Are all the source data underlying the results available to ensure full access and reuse by other researchers?

No source data required

Are the conclusions drawn adequately supported by the data and analysis?

Yes

Is the background of the case’s history and progression described in sufficient detail?

Yes

Reviewer Expertise:

Bioethics, philosophy of medicine, research methodology

I confirm that I have read this submission and believe that I have an appropriate level of expertise to confirm that it is of an acceptable scientific standard.

# Reviewer report: Reviewer response for version 1

**DOI:** 10.21956/openreseurope.18419.r39695

**Published:** 2024-04-30

**Authors:** Tomasz Zuradzki

**Affiliations:** 1 Jagiellonian University, Krakow, Poland

This is a nicely written essay that aims to inspire future interdisciplinary research projects, rather than a scholarly paper.

 The first section describes bioethics as a discipline, most observations are based on common sense or anecdotal evidence (e.g. ‘bioethicists are often invited on big research projects at the last minute’), and this part may benefit from referring to more systematic approaches to the history of bioethics, e.g., presented in Bystranowski et al. (2022)[Ref 1]. The second section refers to human geography and my experience is too limited in this field to propose any changes. The third part is about interdisciplinarity and it is based on the experience of meeting researchers in real life. However, there is no real engagement with abundant work on interdisciplinarity (eg., published in sociology of scientific knowledge), but only very general statements: ‘So, what does it mean to be interdisciplinary? We cannot claim to be interdisciplinary if we pick and choose elements we like the sound or feel of; interdisciplinarity involves sitting with the tensions as well and working with and through them.’ Moreover, I find some sentences not clear enough, e.g.,: ‘Smith writes about the meeting point between radical geography and normative theory, and we believe that this is evidence that there is a need and value to foster intentional interdisciplinarity’. The fact that someone is writing about is not evidence that there is a need and value.

 The first case study on parking spaces provides some interesting observations about things we often ‘take for granted,’ although I’m not sure what exactly is proposed here, beyond very general statements like: “Perhaps, sometimes, bioethics takes these things too much for granted”. The second case study on parkour (defined by the authors as ‘a way of appreciating buildings and structures that are often taken for granted’), maybe because of my limited knowledge of parkour, is incomprehensible for me. Although I appreciate ethical/bioethical aspects of it (e.g., ‘What does public space convey about who is welcomed in a specific society?’), I do not understand the geographical approach, for example, what it means to ‘care for space’ and what is value of such care (‘thinking with parkours as a reclaiming of neglected spaces can make the need for caring for such spaces more salient’). The paper concludes by referring to previous editorial by the authors published in the journal *Bioethics*. In conclusion, the essay may inspire some to think more about the connections between bioethics and human geography, but it would benefit from more precision and examples.

Is the case presented with sufficient detail to be useful for teaching or other practitioners?

Partly

Is the work clearly and accurately presented and does it cite the current literature?

Partly

If applicable, is the statistical analysis and its interpretation appropriate?

Not applicable

Are all the source data underlying the results available to ensure full access and reuse by other researchers?

No source data required

Are the conclusions drawn adequately supported by the data and analysis?

Yes

Is the background of the case’s history and progression described in sufficient detail?

Yes

Reviewer Expertise:

philosophy, bioethics

I confirm that I have read this submission and believe that I have an appropriate level of expertise to confirm that it is of an acceptable scientific standard, however I have significant reservations, as outlined above.

## References

[1] : Half a century of bioethics and philosophy of medicine: A topic-modeling study. *Bioethics* .2022;36(9) : 10.1111/bioe.13087 902-925 10.1111/bioe.13087 36170119 PMC9827984
